# Non-AIDS Mortality Is Higher Among Successfully Treated People Living with HIV Compared with Matched HIV-Negative Control Persons: A 15-Year Follow-Up Cohort Study in Sweden

**DOI:** 10.1089/apc.2018.0015

**Published:** 2018-08-01

**Authors:** Zaake de Coninck, Laith Hussain-Alkhateeb, Göran Bratt, Anna Mia Ekström, Magnus Gisslén, Max Petzold, Veronica Svedhem

**Affiliations:** ^1^ Department of Public Health Sciences, Centre for Global Health, HIV and SRHR, Karolinska Institutet, Stockholm, Sweden.; ^2^ Occupational and Environmental Medicine, Sahlgrenska Academy, University of Gothenburg, Gothenburg, Sweden.; ^3^ Department of Clinical Science and Education, Stockholm South General Hospital, Stockholm, Sweden.; ^4^ Department of Infectious Diseases/Venhälsan, Stockholm South General Hospital, Stockholm, Sweden.; ^5^ Department of Infectious Diseases, Karolinska Institutet, Karolinska University Hospital, Stockholm, Sweden.; ^6^ Department of Infectious Diseases, Sahlgrenska Academy, University of Gothenburg, Gothenburg, Sweden.; ^7^ Health Metrics at Sahlgrenska Academy, University of Gothenburg, Gothenburg, Sweden.

**Keywords:** HIV, ART, mortality, successful treatment, viral load, viral suppression

## Abstract

There is an ongoing debate whether the life span of successfully treated people living with HIV (PLHIV) is comparable with that of the general population. The aim of this cohort study is to compare all-cause mortality between all PLHIV, successfully treated PLHIV, and HIV-negative control persons from the general population and to explore the impact of viral load (VL) at diagnosis. A total of 4066 PLHIV were matched against 8072 HIV-negative controls according to age, sex, and region of birth. Further, associations between VL at diagnosis, time on treatment, treatment outcome, and mortality were assessed over a 15-year period. Cox regression estimates were computed to compare the overall crude and adjusted hazard ratios (HRs) for mortality. After a 15-year follow-up period, successfully treated PLHIV were found to be three times more likely to die when compared with HIV-negative controls (HR 3.01, 95% CI 2.05–4.44, *p* < 0.001). The risk of mortality decreased from HR 6.02 after the first year of successful treatment. VL >30,000 c/mL at diagnosis was associated with an increased risk of mortality despite long-term antiretroviral therapy (ART) treatment. Although effective viral suppression has led to significant increases in longevity and quality of life, ART has not fully restored life expectancy to a level comparable with that found in HIV-negative persons. Even when PLHIV are successfully treated, there are several other important areas related to death, such as smoking and social factors, where data are still missing.

## Introduction

Antiretroviral therapy (ART) has significantly reduced mortality and morbidity among people living with HIV (PLHIV).^1^ This trend will continue to improve as recently concluded studies (2017) suggest that the more recently the patients are treated, the lower their risk of mortality (due to access to better drugs, better treatment and preventative measures, and better management of comorbidities).^2,3^ However, although some researchers have suggested that the life span of successfully treated PLHIV is now comparable with that of the general population,^4^ others continue to find that morbidity and mortality rates of PLHIV remain elevated when compared with HIV-negative persons.^5–7^

Immunodeficiency because of HIV infection has been presented as a major risk factor.^8–10^ One major European study conducted in 2012 followed 35,000 ART-treated PLHIV with the CD4^+^T cell count (CD4) >500 cells/μL over a mean period of 3.5 years^11^ and found mortality rates among PLHIV, excluding people who inject drugs (PWID), to be similar to the rates found in the general population (0.37/100 PY). One other study had similar findings, although with ART-treated PLHIV with a baseline CD4 > 350.^12^ These studies are some of many that have found CD4 to be strongly predictive of mortality. Further, the robust START study published in 2015 highlighted net differences in serious events and all-cause mortality between those who started treatment with a high CD4 (>500 cells/μL) compared with those who delayed treatment start until CD4 had dropped to 350 cells/μL.^13^ The immediate start of treatment was found to significantly reduce the rate of both AIDS and non-AIDS events. Since many HIV-positive persons present late for care with a very low CD4, the implications are important.^14,15^ The impact of viral load (VL) at diagnosis on morbidity and mortality has been well documented,^16^ but there were few clinical data concerning the impact of VL at diagnosis on mortality following long-term treatment.

Research on the topic of mortality rates among PLHIV is characterized by an inconsistency in findings due to the following major limitations: (a) small sample sizes; (b) the use of the general population (characterized by its ever-increasing life expectancy) as a comparison group, as opposed to using matched HIV-negative controls (which would allow for stratifying by potential risk factors); (c) limited follow-up periods; and (d) lack of data concerning social factors. Sweden's National Quality Assurance Register (InfCareHIV) was used as the data source in this study. Over 99% of HIV patients in Sweden (including migrants) are monitored from diagnosis until death or migration through InfCareHIV, a database that has been implemented in all HIV care centers in the country. In Sweden, 90% of the InfCareHIV cohort is considered to be successfully treated,^17^ and the country achieved the UNAIDS 90-90-90 target in 2014, with 90% of HIV patients knowing their status, 94.94% receiving sustained ART, and 94.53% of patients on ART having suppressed their VL. Data collected from this cohort study will allow us to contribute robust evidence to this field of research.

The main aim of this study is to compare all-cause mortality between all PLHIV and matched HIV-negative controls and to explore the impact that VL at HIV diagnosis has on mortality rates. A further objective of the study is to compare all-cause mortality between successfully treated PLHIV and HIV-negative controls.

## Materials and Methods

### Study setting and data collection

Patients were identified from the Swedish National HIV Register (InfCareHIV) that includes >99% of patients living in Sweden (including migrants with and without residence permits). Patients are enrolled into the register at the time of HIV diagnosis. InfCareHIV collects sociodemographic, biological, and treatment data at least every 6 months. This study population included 4066 PLHIV who had been diagnosed with HIV in Stockholm or Gothenburg between 1996 (the year when effective ART was introduced into the Swedish public health system) and 2011 and from the rest of the country between 2005 and 2011. All patients have a national registration number (NRN). The NRN enabled database linkages. Persons who had been identified in the InfCareHIV database, but did not have an NRN, were excluded from this study (414 patients in total). PLHIV who had been diagnosed outside of Sweden were also excluded (additional 400 patients) due to their lack of care and treatment history.

The Swedish Population Register (SPR) carries records on all individuals residing in Sweden on a permanent basis. Statistics Sweden (www.scb.se Microdata at Statistics Sweden for research purposes) matched the 4066 PLHIV from InfCareHIV with 8072 HIV-negative persons from SPR. Mortality was determined using the Cause of Death Register (www.socialstyrelsen.se/english) that includes date and cause of death coded according to the international version of the disease classification system (ICD9–ICD10). The World Health Organization (WHO) and Centers for Disease Control (CDC) system of classification was used for AIDS diagnosis. Today, 20% of the Swedish HIV cohort are current smokers (InfCareHIV unpublished result), but at the study time, 1996–2011 smoking and educational statuses were not collected.

### Ethical clearance

All the above-mentioned registries are protected by strict confidentiality agreements, but may be disclosed for research purposes. All data were anonymous and the Regional Ethics Committee in Stockholm approved the use of these data for the purpose of this study (Diary No. 2012/1187-31/5).

### Data matching

**FIG. 1.**
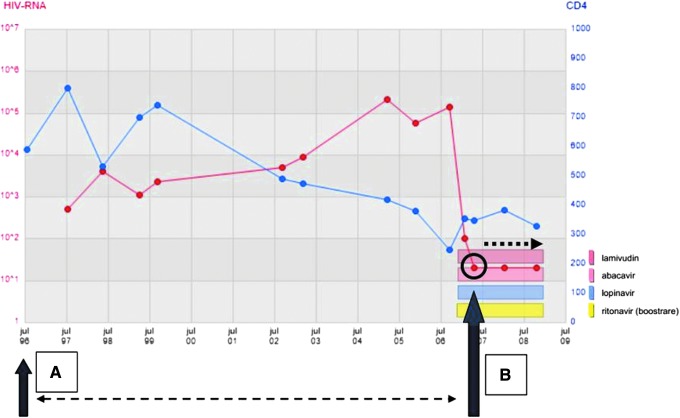
Inclusion criteria for the substudy. A, time point at which a person is diagnosed as HIV+. B, VL < 500 HIV-1 RNA c/mL within 6 months after ART initiation.

Each HIV-positive person (hereafter referred to as an HIV+ person) was matched to two HIV-negative persons (hereafter referred to as control persons) by age at the year of HIV diagnosis. Exact matching to control person was carried out according to (a) year of birth, (b) sex (male; female), and (c) region of birth using the UNAIDS classification system with the addition of Sweden as a distinct category (Sweden; Europe, USA, Israel, and Canada; sub-Saharan Africa; North Africa and the Middle East; Eastern Europe and Central Asia; Asia and the Pacific; and the Caribbean and Latin America). Marker A in Fig. 1 illustrates the time point at which a person is diagnosed as HIV+ and is matched to an HIV-negative control person.

### Substudy: comparing successfully treated PLHIV and HIV-negative persons

To explore how successful treatment impacts mortality, we compared mortality between the successfully treated PLHIV and HIV-negative control persons. PLHIV with a VL that had dropped to <500 HIV-1 RNA c/mL within 6 months after ART initiation were included in this substudy. This is illustrated as time point B in Fig. 1. Persons whose VL had dropped to <500 c/mL at a later stage were not included. At this start of follow-up point, we ensured that we had at least one control person for each HIV+ person, otherwise the HIV+ person was removed from the analysis. Follow-up duration was defined as the number of days that an HIV+ person was able to keep the VL <500 c/mL. HIV+ persons ceased to be followed up: (a) at death or at loss to follow-up (e.g., migration); (b) the moment VL ≥500 c/mL; and (c) at the end of the study (end of 2011). The patients in this cohort were treated according to the Swedish HIV treatment guidelines that are, for the most part, consistent with the European guidelines (EACS and BHIVA).

### Statistical methods

Background and characteristics were described at baseline for both groups (HIV+; HIV-negative control person): sex, age, and region of birth. Comparing these indicators between the two groups allowed us to verify the success of the matching process. Age at death (in years), follow-up time from diagnosis (in days), and mortality rates/100 P-Y were also compared using *t*-tests.

Cox regression estimates were computed to compare the overall crude hazard ratio (HR) for mortality (yes; no) between successfully treated PLHIV and HIV-negative control persons over a 15-year period. To understand the role played by time, we also compared the HR for mortality for five additional successive follow-up periods (1 year, 3 years, 4 years, 5 years, and 10 years).

Among PLHIV, we compared the following indicators using a chi-square test: CD4 at HIV diagnosis <200/μL (yes; no), VL level at HIV diagnosis ≥30000 c/mL (yes; no), and mode of transmission (heterosexual; MSM; PWID; and other).

VL level at HIV diagnosis <30,000 c/mL (yes; no) and HIV-1 RNA copies <500 c/mL within 6 months of the start of ART throughout 15 years of follow-up (yes; no) were modeled using Cox regression to compare their impact on risk of mortality among PLHIV. The latter indicator was used as a proxy reading for those who were successfully treated. We used Akaike Information Criteria (AIC) to compare different models in the multiple Cox regression analysis. AIC is a statistical test based on the log-likelihood function to assess the best-fit model. A smaller AIC value indicates a better-fitted model.

Data were analyzed using STATA, version 14.0 (College Station, StataCorp LP, TX). In both stages of the analysis, 500 HIV-1 RNA c/mL was used as the cutoff value since our preferred value (50 c/mL) was only implemented in Sweden in 1998, 2 years after we began to collect data on HIV+ persons. In addition, a cutoff value of 500 HIV-1 RNA c/mL prevented us from excluding patients who exhibited sudden and temporary spikes in viremia (viral blips).

## Results

### Baseline and matching characteristics

A total of 4066 PLHIV were matched against 8072 HIV-negative controls (Table 1), giving a ratio of 1 case to 2 controls. Males and females were equally distributed between the two groups. In total, 38% of the participants originated from Sweden, 34% from sub-Saharan Africa, 10% from Asia and the Pacific, 8% from Europe, USA, Israel, and Canada, and the remainder originated from other parts of the world. Despite exact matching, marginal differences between the groups were observed due to occasional dropout.

**Table 1.** Baseline Characteristics: Comparing PLHIV and HIV-Negative Persons

*Baseline characteristics*	*HIV positive (%),* n* = 4066*	*HIV negative (%),* n* = 8072*	*Total,* n* = 12,138*
Sex, *n* (%)			
Male	2605 (64.07)	5184 (64.22)	7789 (64.17)
Female	1461 (35.93)	2888 (35.78)	4349 (35.83)
Age, years, mean (SD)	37.60 (11.23)	37.60 (11.23)	37.60 (11.23)
Region of birth, *n* (%)			
Sweden	1526 (37.53)	3052 (37.81)	4578 (37.72)
Europe, USA, Israel, and Canada	316 (7.78)	631 (7.82)	947 (7.80)
Sub-Saharan Africa	1373 (33.76)	2697 (33.41)	4070 (33.53)
North Africa and Middle East	87 (2.31)	174 (2.16)	261 (2.15)
Eastern Europe and Central Asia	168 (4.13)	334 (4.14)	502 (4.14)
Asia and the Pacific	397 (9.76)	791 (9.80)	1188 (9.79)
The Caribbean and Latin America	199 (4.89)	393 (4.87)	592 (4.88)

### Comparing mortality between PLHIV and HIV-negative control persons

During the 15-year study period, 275 (6.76%) PLHIV died compared with 110 (1.36%) HIV-negative controls. The mean age at death was 50.54 years for PLHIV and 56.84 years for HIV-negative persons (Table 2).

**Table 2.** Comparing Mortality PLHIV and HIV-Negative Persons

	*HIV positive,* n* = 275/4066*	*HIV negative,* n* = 110/8072*	p	*Total,* n* = 385/12,138*
Age (years) at death, mean (SD)	50.54 (12.7)	56.84 (13.6)	<0.001	52.33 (13.2)
Follow-up period from diagnosis (days), mean (SD)	2196 (1551)	2304 (1585)	<0.001	2267 (1575)
Mortality rates/100 PY (95% CI)	1.13 (1.00–1.27)	0.22 (0.18–0.26)	<0.001	0.51 (0.46–0.56)

CI, confidence interval.

### Mortality among PLHIV

At diagnosis, 55.16% of all PLHIV had a VL ≥30,000 c/mL and 29.26% had a CD4 < 200 (Table 3). CD4 < 200 and VL ≥30,000 c/mL at diagnosis were both significantly linked to an increased risk of mortality (*p* < 0.001). Of the 275 deaths among PLHIV, 107 (38.91%) had CD4 < 200 at diagnosis compared with 1083 (28.57%) of those who were still alive (*p* < 0.001). Among the 275 who died, the initial VL was ≥30,000 c/mL among 171 of the patients (62.18%) and <30,000 c/mL among 83 of the patients (30.18%) (Table 3).

**Table 3.** Risk Factors for Mortality Amongst PLHIV

	*Alive,* n* = 3791*	*Deceased,* n* = 275*	p	*Total,* N* = 4066*
CD4^+^T cells <200 at diagnosis, *n* (%)				
Yes	1083 (28.57)	107 (38.91)	0.001	1190 (29.26)
No	2690 (70.96)	151 (54.91)		2841 (69.87)
Missing	18 (0.47)	17 (6.18)		35 (0.86)
VL ≥30,000 c/mL at diagnosis, *n* (%)				
Yes	2072 (54.66)	171 (62.18)	0.001	2243 (55.16)
No	1692 (44.63)	83 (30.18)		1775 (43.65)
Missing	27 (0.71)	21 (7.64)		48 (1.18)
Mode of transmission, *n* (%)				
Heterosexual	2067 (54.52)	104 (37.82)	0.001	2171 (53.39)
MSM	1147 (30.26)	51 (18.55)		1198 (29.46)
PWID	243 (6.41)	74 (26.91)		317 (7.80)
Other	221 (5.83)	29 (10.55)		250 (6.15)
Missing	113 (2.98)	17 (6.18)		130 (3.20)

MSM, men who have sex with men; PWID, people who inject drugs; VL, viral load.

**FIG. 2.**
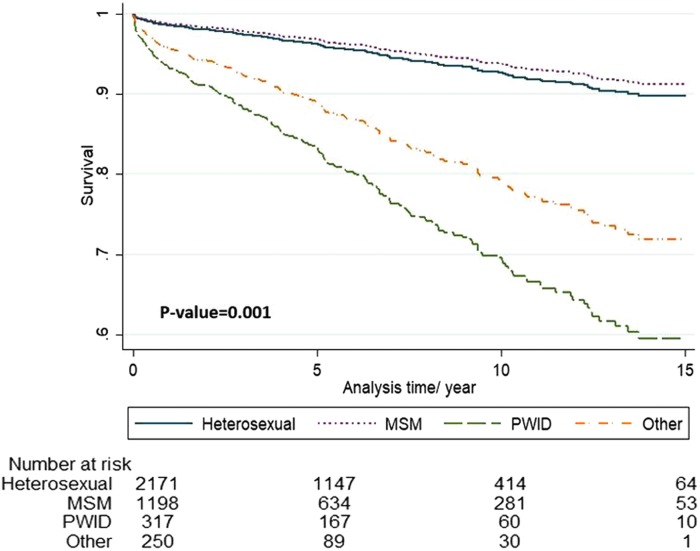
Mortality among PLHIV (mode of transmission; heterosexual, MSM, PWID, and other). MSM, men who have sex with men; PLHIV, people living with HIV; PWID, people who inject drugs.

The mode of HIV transmission among persons who were still alive was primarily heterosexual (54.52%), followed by men who have sex with men (MSM) (30.25%), and PWID (6.41%). The mode of HIV transmission among deceased PLHIV was primarily heterosexual (37.82%), followed by PWID (26.91%), and then MSM (18.55). These findings were all significant (*p* < 0.001) (Fig. 2). At diagnosis, there were 35 missing records from CD4, 48 from VL analysis, and 130 missing records concerning the mode of transmission (113 among those still alive and 17 among the deceased) (Table 3).

Most missing data from deceased patients are from 1996 to 1998 on patients diagnosed close to death or postmortem. The three most common non-AIDS-related diagnoses at death were cancer (non-AIDS related): 56 persons; CHD/CVD/VD: 50 persons; and accident/suicide/drug overdose: 36 persons.

### Risk factors for mortality among PLHIV

**FIG. 3.**
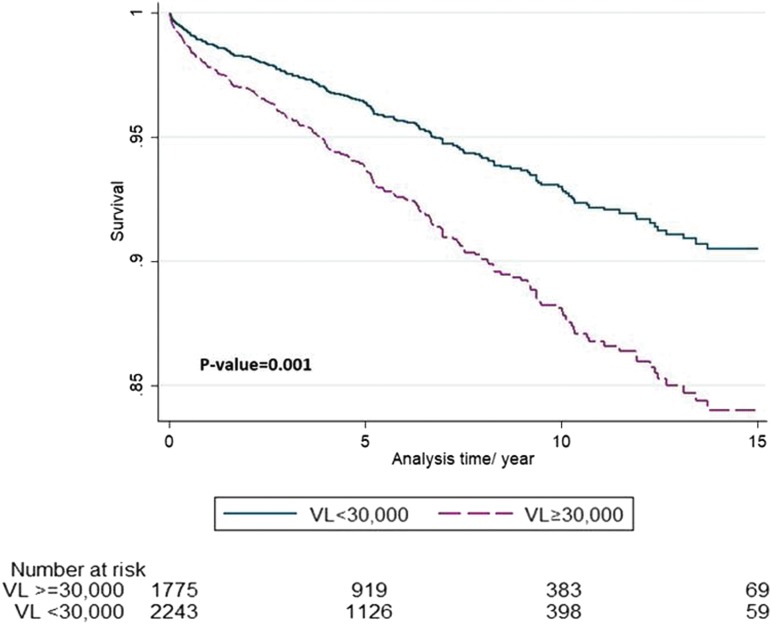
Mortality among PLHIV (VL ≥30,000 vs. VL <30,000 at diagnosis). PLHIV, people living with HIV; VL, viral load.

Throughout the population of PLHIV (*n* = 4066), Cox regression revealed that VL ≥30,000 c/mL at diagnosis was associated with a risk of mortality 1.74 times greater than patients with VL <30,000 c/mL at diagnosis (*p* < 0.001) (Fig. 3). After adjusting for patients with CD4 < 200 cells/μL at diagnosis, the risk of mortality dropped to 1.56 (1.18–2.06). Adjusting for death due to alcohol, narcotics, and suicide, the hazard of death for VL ≥30,000 dropped, but remained significant (HR = 1.39; 95% CI 1.06–1.84). After also adjusting for CD4 < 200 cells/μL, the risk of mortality was ∼1.96 times greater for VL ≥30,000 c/mL than VL <30,000 c/mL at diagnosis (HR = 1.56; *p* < 0.005), and after adjusting for age, sex, and nationality, the risk of mortality was less, 1.35 (*p* < 0.05) (Table 4).

**Table 4.** Crude and Adjusted Cox Regression Analysis Comparing Mortality Among Patients with VL ≥30,000 vs VL <30,000 at Diagnosis

*Predictors*	*HR (95% CI)*				
	*Crude model*	*Adjusted model 1*	*Adjusted model 2*	*Adjusted model 3*	*Adjusted model 4*
VL, c/mL					
<30,000	Ref.	Ref.	Ref.	Ref.	Ref.
≥30,000	1.74 (1.34–2.26)	1.39 (1.06–1.84)	1.96 (1.48–2.47)	1.35 (1.04–1.76)	1.56 (1.18–2.06)

Model 1: adjusted for patients' mortality due to alcohol consumption, narcotics, and suicide.

Model 2: adjusted for patients' mortality due to alcohol consumption, narcotics, suicide, and CD4 (<200 cells/μL) at diagnosis.

Model 3: adjusted for patients' age, sex, and nationality.

Model 4: adjusted for patients' CD4 (<200 cells/μL) counts at diagnosis.

CI, confidence interval; HR, hazard ratio; VL, viral load.

### Comparing mortality between successfully treated PLHIV and HIV-negative control persons

Overall, after 15 years of follow-up, successfully treated PLHIV were found to be three times more likely to die compared with HIV-negative controls (HR 3.01, 95% CI 2.05–4.44, *p* < 0.001) (Table 4). During the first year of follow-up, PLHIV were six times more likely to die when compared with HIV-negative controls (HR 6.02, 95% CI 2.39–15.20, *p* < 0.001). After 3 years of follow-up, PLHIV were 3.5 times more likely to die than HIV-negative controls (HR 3.48, 95% CI 2.04–5.91, *p* = 0.001). After 4 years of follow-up, PLHIV were 3.4 times (HR 3.44, 95% CI 2.14–5.52, *p* < 0.001) more likely to die than HIV-negative controls, and after 5 years of follow-up, they were 3.5 times more likely to die (HR 3.45, 95% CI 2.19–5.42, *p* = 0.001). During the first 10 years of follow-up, PLHIV were three times (HR 2.96, 95% CI 1.99–4.41, *p* = 0.001) more likely to die than HIV-negative controls. Throughout the 15-year follow-up period, PLHIV exhibited markedly lower survival rates than HIV-negative persons (Table 4). Adjusting for potential confounders such as age, sex, origin, and calendar time of diagnosis did not reveal any significant change in the association at any follow-up period. Conducting a sensitivity analysis stratifying for drug users, non-PWID and PLHIV were still 2.4 times more likely to die than HIV-negative persons (HR = 2.41; *p* < 0.001) (Table 5).

**Table 5.** Cox Regression Comparing Mortality Between Successfully Treated PLHIV and HIV-Negative Persons

*Follow-up period*	*Cohort*	*People at risk*	*Deceased*	*Crude HR*	
				*HR (95% CI)*	p
Entire period (15 years)	HIV−	4553	47	Ref.	<0.001
	HIV+	2297	58	3.01 (2.05–4.44)	
Ten years	HIV−	598	44	Ref.	<0.001
	HIV+	162	55	2.96 (1.99–4.41)	
Five years	HIV−	1607	31	Ref.	<0.001
	HIV+	580	48	3.45 (2.19–5.42)	
Four years	HIV−	2018	28	Ref.	<0.001
	HIV+	789	44	3.44 (2.14–5.52)	
Three years	HIV−	2495	22	Ref.	<0.001
	HIV+	1043	36	3.48 (2.04–5.91)	
One year	HIV−	3845	6	Ref.	<0.001
	HIV+	1836	18	6.02 (2.39–15.2)	

CI, confidence interval; HR, hazard ratio.

A cause-specific hazard analysis was conducted to compare non-AIDS mortality rates between successfully treated PLHIV and HIV-negative controls. Only 11 of 58 patients in our cohort died from an AIDS-related condition. When AIDS-attributed mortality is excluded, successfully treated PLHIV are still 2.4 times more likely to die compared with HIV-negative persons (HR 2.43, 95% CI 1.61–3.65, *p* < 0.001).

### AIDS-related deaths in successfully treated PLHIV

AIDS-related mortality after the first year of successful ART was primarily ascribed to lymphoma (Table 6).

**Table 6.** Patients Dying from AIDS According to WHO and CDC Classification Systems

*Days of successful treatment before AIDS diagnosis*	*AIDS condition (CDC classification)*
36	Cryptococcosis, extrapulmonary
65	Lymphoma, Burkitt
115	Encephalopathy, HIV-related
183	Kaposi sarcoma
252	Bacterial pneumonia, recurrent
301	Histoplasmosis
377	Lymphoma, Burkitt
605	Lymphoma, Burkitt
771	Aids, not specified
1101	Lymphoma, Burkitt
1178	Cryptococcosis, extrapulmonary

CDC, Centers for Disease Control.

### Comparing the survival rate between successfully treated PLHIV and untreated or unsuccessfully treated patients

**FIG. 4.**
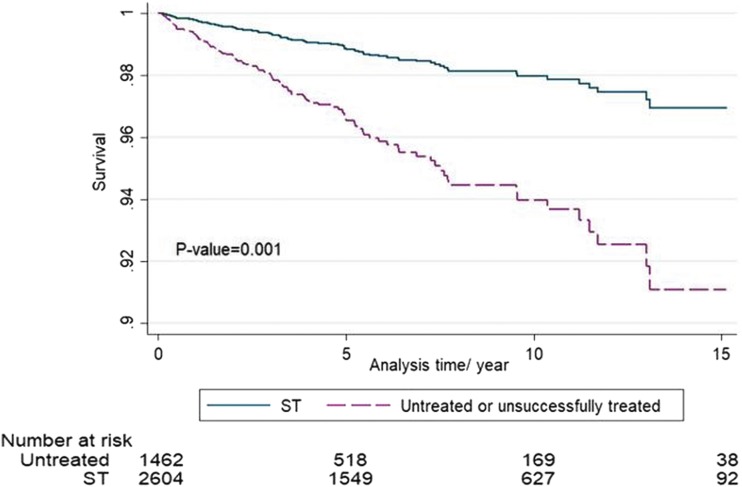
Mortality between successfully treated PLHIV and untreated or unsuccessfully treated PLHIV. PLHIV, people living with HIV.

Additionally, successfully treated PLHIV, *n* = 2604 (64.04%), had a 76% reduced risk of mortality compared with untreated or unsuccessfully treated HIV patients over a period of 15 years (HR = 0.24, 95% CI 0.19–0.31, *p* = 0.001) (Fig. 4). The risk of mortality is still reduced after adjusting for other potential factors such as mode of transmission, year of diagnosis, and VL at diagnosis (HR = 0.37; *p* = 0.001). This HR becomes 0.45; *p* < 0.001 when we also add CD4 as a possible confounder in the model.

## Discussion

The major finding of this study is the significantly higher all-cause mortality observed among successfully treated PLHIV when compared with HIV-negative control persons. Despite being virologically suppressed, successfully treated PLHIV were still 3 times more likely to die compared with HIV-negative controls. The mean age at death was lower among PLHIV (50.54 years) compared with HIV-negative controls (56.84 years) with a difference of 6.30 years. This finding is similar to those published by other authors^18,19^ and suggests that although life expectancy has improved considerably due to advancements in care and treatment, a significant gap remains compared with the general population.

The impact that VL at diagnosis has on morbidity and mortality has been well documented,^16^ but there is little clinical data on the impact that a high VL at diagnosis has on mortality after long-term treatment. We found an increased risk of mortality (HR = 1.74) for those with VL ≥30,000 at diagnosis even after adjusting for CD4 at diagnosis (HR = 1.56). This increased risk of mortality for VL ≥30,000 c/mL at diagnosis might have been lower if this cohort had included participants up to 2016; currently, >90% of InfCareHIV patients have sustained viral suppression compared with the 64% (*n* = 2604) in our cohort. This hypothesis is supported by our observation that mortality is reduced by 76% among successfully treated compared with untreated and unsuccessfully treated PLHIV. This has implications from the care perspective; patients with a history of treatment failure and VL ≥30,000 c/mL at diagnosis might have a higher risk of mortality and need closer contact with the healthcare system.

The increased risk of mortality after 3 and 4 years in this study (HR = 3) contrasts with one other study conducted in North America, where PLHIV on ART with a baseline CD4 > 350 had mortality rates comparable with the general population.^12^ It also contrasts with the COHERE 2012 study that followed PLHIV on ART (CD4 ≥ 500) over a median period of 3.5 years and found mortality rates similar to that of the general population.^11^ However, the COHERE study had also found that mortality was 4.2 times higher than in the general population when all PLHIV (regardless of CD4 and mode of transmission) were considered. The difference between our findings and the COHERE study may be due to Sweden being a low-endemic country with a Communicable Diseases Act that favors patients who adhere to care, and where care and ART are provided free of charge to PLHIV, it may achieve lower mortality among the HIV cohort. However, it may also be due to the study design; mortality among HIV infected in Sweden is too low to affect the mortality rate in the general population. The HIV cohort with 62.54% immigrants, as opposed to 18.56% immigrants in the general population, was matched for region of birth. This avoids overestimation of demographic-related mortality rates.

Equally significant in our study is the following steady trend: the longer that successfully treated PLHIV survive, the lower their risk of mortality compared with matched HIV-negative control persons. During the first year of follow-up, HIV+ persons were six times more likely to die than HIV-negative persons. After 10 years, PLHIV were only three times more likely to die compared with HIV-negative persons. Although the difference is still present after 15 years of follow-up, we speculate that it might decrease even more after 20 or 30 years. Studies conducted in Europe and North America in 2017 highlight the fact that the more recently a patient is enrolled in the healthcare system (and consequently has access to less toxic drugs, improved adherence, prophylactic measures, and management of comorbidity), the lower their risk of mortality.^2,3^ We thus also speculate that the improvements associated with healthcare developments are going to continue to improve survival rates.

When we conducted a sensitivity analysis stratifying for drug use, we found that PLHIV, non-PWID, and successfully treated were still 2.4 times more likely to die than HIV-negative persons. This strengthens our findings, although the ratio is lower than the aforementioned calculation, which includes all HIV+ persons. One possible explanation for this small difference could be that many PWID in the study keep their VL <500 c/mL and were no longer active users of drugs (InfCareHIV unpublished result).

Successfully treated PLHIV are still 2.5 times more likely to die from non-AIDS causes than HIV-negative persons. Immune activation, chronic low-grade inflammation, coagulation disorders, and/or lipid disturbances,^20^ as well as lifestyle risk factors such as smoking,^21^ continue to cause this increased mortality. In addition, the long-term use of earlier nucleoside reverse transcriptase inhibitors and first-generation protease inhibitors inducing toxicities and metabolic disturbances may further increase the risk of mortality for PLHIV.^22–24^

### Strengths and limitations

A major strength of this study is that it addresses limitations faced by several previous studies on this topic. First, the coverage of our data is high thanks to the National Quality Assurance Registry that includes information on >99% of PLHIV in Sweden, which means that people diagnosed postmortem are also included. Second, the sample size in this study is considerable (further demonstrated by the significant statistical power even within each of the follow-up periods), as is the 15-year follow-up period. Further, appropriate HIV-uninfected comparison groups were used. The unique Swedish situation mentioned above makes the generalization to PLHIV in other countries uncertain.

The limitation is that data concerning several other important areas related to death, such as social factors, were missing. In addition, we did not have access to data on smoking during follow-up. We know from the health questionnaire in InfCareHIV used in 2017 that 20% of the HIV-infected people in Sweden were smokers. Given that for mortality the HR is 1.8 for smokers versus nonsmokers, we would expect about 14% of the mortality in our study to be caused by smoking. Thus, smoking cannot entirely explain the observed results in this study.

InfCareHIV utilizes VL suppression after 6 months from ART initiation as a determinant for treatment success, whereas WHO uses 48 weeks as the time frame.^25^ An alternative system has recently been suggested, which is the proportion of time while undergoing treatment that a person fully suppresses VL (%FS).^26^ These different systems highlight the fact that best research practices have yet to be agreed upon. Several considerations remain: if a patient suffers from one temporary spike in VL or a rise in VL due to developing viral resistance, does one reset the follow-up period and choose the longest continuous follow-up period of viral suppression or does the researcher add the first and second periods at the individual or cohort level?^27^ In the present study, we adhered to the most common method and only used the first continuous successful treatment period. However, we realize that it is of importance to improve on this methodological approach.

In conclusion, the risk of mortality decreases with each passing year over a 15-year period after 1 year of successful treatment, but PLHIV continue to exhibit a risk of mortality three times higher than HIV-negative persons.
